# The effectiveness of digital solutions in improving nurses’ and healthcare professionals’ mental well-being: a systematic review and meta-analysis

**DOI:** 10.1177/17449871241226914

**Published:** 2024-05-20

**Authors:** Tiina Ilola, Mikael Malmisalo, Elina Laukka, Heli Lehtiniemi, Tarja Pölkki, Maria Kääriäinen, Hong-Gu He, Outi Kanste

**Affiliations:** Nurse Manager, Research Unit of Health Sciences and Technology, University of Oulu, Oulu, Finland; MSc Student, Research Unit of Health Sciences and Technology, University of Oulu, Oulu, Finland; Post-doctoral Researcher, Research Unit of Health Sciences and Technology, University of Oulu, Oulu, Finland; Statistician, Infrastructure for Population Studies, University of Oulu, Oulu, Finland; Professor, Research Unit of Health Sciences and Technology, University of Oulu, Oulu, Finland; Professor, Research Unit of Health Sciences and Technology, University of Oulu, Oulu, Finland; Professor, Alice Lee Centre for Nursing Studies, National University of Singapore, Singapore, Singapore; Professor, Research Unit of Health Sciences and Technology, University of Oulu, Oulu, Finland

**Keywords:** digital health, digital solutions, effectiveness, healthcare professionals, mental well-being, nurse, systematic review, work engagement

## Abstract

**Background:**

Widespread challenges to mental well-being among nurses and healthcare professionals threaten the productivity and quality of healthcare. Digital solutions may prove to effectively support nurses’ and healthcare professionals’ mental well-being.

**Aim:**

To synthesise evidence regarding the effectiveness of digital solutions in improving nurses’ and healthcare professionals’ mental well-being.

**Methods:**

This systematic review followed the JBI guidance for systematic reviews of effectiveness. The PubMed, CINAHL, Scopus, Pro-Quest and APA PsycArticles databases were reviewed for randomised controlled trials and quasi-experimental studies published at any point prior to the 26th of October 2021. Meta-analysis and narrative synthesis were performed.

**Results:**

Fourteen studies were included. Personal mental well-being solutions significantly improved nurses’ and healthcare professionals’ mental well-being. The effectiveness of work-related digital solutions could not be demonstrated. The meta-analysis revealed little to no effect on professionals’ work engagement.

**Conclusions:**

Personal digital solutions may have the potential to improve the mental well-being of nurses and healthcare professionals. With the support of nurse managers’ facilitation, nurses have a key role to promote their own mental well-being by utilising digital mental health solutions. Nevertheless, further adequately powered, well-designed research is required.

## Introduction

Mental health disorders have become common among nurses and healthcare professionals (Marvaldi et al., 2021; Ye, 2021). Methods that target the mental well-being of professionals should be introduced as mental health disorders can lower productivity and weaken the quality of healthcare (Marvaldi et al., 2021). The interaction between mental health, work environment and technology must be understood to sufficiently support the mental well-being of nurses and healthcare professionals. Innovative solutions are needed to mitigate the psychological effects of crises such as the COVID-19 pandemic (WHO, 2022a; Ye, 2021).

Mental health is defined as a state of well-being. Well-being can be divided into mental, social, physical and spiritual well-being, activities and functioning and personal circumstances. Mental well-being helps an individual cope with challenges, realise their abilities, make decisions, build relationships and work effectively (Ackerman et al., 2018; WHO, 2022b). Work engagement is significantly related to job satisfaction and is one of the most positive outcomes identified by an organisation (Yildiz and Yildiz, 2022). This aspect of work, which is promoted by employees’ personal self-efficacy and resilience, positively impacts nurses’ retention, patient satisfaction and nursing outcomes (Porter and Wang, 2022). Well-being has various definitions, intensities and outcomes (Ackerman et al., 2018; WHO, 2022b). A wide range of scales for measuring life-enhancing and life-depleting constructs exists in the field of positive psychology. These scales measure either positive (e.g. general well-being) or negative (e.g. stress) outcomes. They also enable researchers to explore positive mental responses to adverse situations (Ackerman et al., 2018). In the present systematic review, we consider mental well-being broadly in the sense that it includes aspects of well-being at work (e.g. work engagement).

Nurses and healthcare professionals have taken advantage of various digital solutions to maintain the quality of their clinical work (Drissi et al., 2021). In this systematic review, we consider digital solutions to be an umbrella term for different electronic, mobile or virtual technologies, software or applications, which are delivered in addition to, or as a substitute for, traditional models. Digital solutions can be personal, work-related or educational. In nursing and healthcare, digital solutions are used for patient care, education, evidence-based services and personal well-being. According to the systematic review by Nguyen et al. (2021), personal digital literacy seems to be a key determinant in improving nurses’ well-being.

A preliminary search of the Cochrane Database of Systematic Reviews, PubMed and JBI Evidence Synthesis did not return any prior systematic reviews concerning the effectiveness of digital solutions to improve nurses’ and healthcare professionals’ well-being. According to previous systematic reviews, mobile applications have been used to mitigate burnout, depression and suicidality among nurses and healthcare professionals (Pospos et al., 2018), as well as support their communication and management of care (Gonçalves-Bradley et al., 2020). Web-based psychological interventions have been shown to be effective at supporting psychological well-being (Carolan et al., 2017; Gál et al., 2021), perceived stress, depression (Gál et al., 2021) and work effectiveness (Carolan et al., 2017) in general populations. Even though the current evidence base is rather weak, there is a growing impression that digital solutions may play a key role in providing effective support for the mental well-being of nurses and healthcare professionals (Ye, 2021). Healthcare requires effective strategies for both improving the job performance of professionals and optimising the quality of care (Dutton and Kozachik, 2020; Guo et al., 2020).

The aim of this systematic review was to synthesise the evidence of the effectiveness of digital solutions in improving nurses’ and healthcare professionals’ mental well-being. The review questions were:

1. What kinds of digital solutions are used to improve nurses’ and healthcare professionals’ mental well-being?
2. Which mental well-being outcomes do the digital solutions used by nurses and healthcare professionals affect?
3. How effective are digital solutions in improving the mental well-being of nurses and healthcare professionals?

## Methodology

This systematic review followed the JBI methodology for systematic reviews of effectiveness (Tufanaru et al., 2020) and the PRISMA statement (Page et al., 2021). The inclusion criteria were formulated according to the PICOS format (Table 1).

**Table 1.** The inclusion criteria of this systematic review of effectiveness of digital solutions improving healthcare professionals’ mental well-being were formulated according to the PICOS format. The search strategy included Medical Subject Headings (MeSH) of digital solutions, mental well-being, and healthcare professionals. Five databases (PubMed, CINAHL, Scopus, Pro-Quest and APA PsycArticles) were reviewed for research published up to 26 October 2021.

PICOS format	Inclusion criteria	Exclusion criteria
Participants	Healthcare professionals: nurses; doctors and allied healthcare staff (e.g. pharmacists and therapists).	Non-professionals, caregivers, patients or clients, family members, students, trainees, residents, undergraduates, teachers, educators, leaders, managers
Intervention	Digital solution (various IT and virtual mobile applications or software which are delivered in addition to, or as substitute for, the traditional model). Personal or work-related intervention	Non-digital solutions Educational intervention
Comparison intervention	Non-digital (traditional or manual model) intervention No intervention	No comparator
Outcomes	Outcomes describing positive and negative mental well-being (such as general well-being, self-efficacy, stress, or burnout) or mental well-being at work (such as job satisfaction, work engagement or job performance)	Outcomes describing mental well-being or mental well-being at work not assessed
Types of studies	Randomised controlled trials Quasi-experimental trials	Other study designs Review
Publication details	Original, peer-reviewed study English Full-text available	Protocol, ongoing study, dissertation, journal article, conference paper, report, editorial, letter, news, book, promotional or advertising article

### Search strategy

The search strategy included Medical Subject Headings (MeSH) of digital solutions, mental well-being, and healthcare professionals (Supplemental Table S1). Five databases (PubMed, CINAHL, Scopus, Pro-Quest and APA PsycArticles) were reviewed for research published up to 26 October 2021. The search was supplemented by a manual search of the reference lists of the included studies and a search for grey literature in the BASE database.

### Study selection

**Figure 1.**
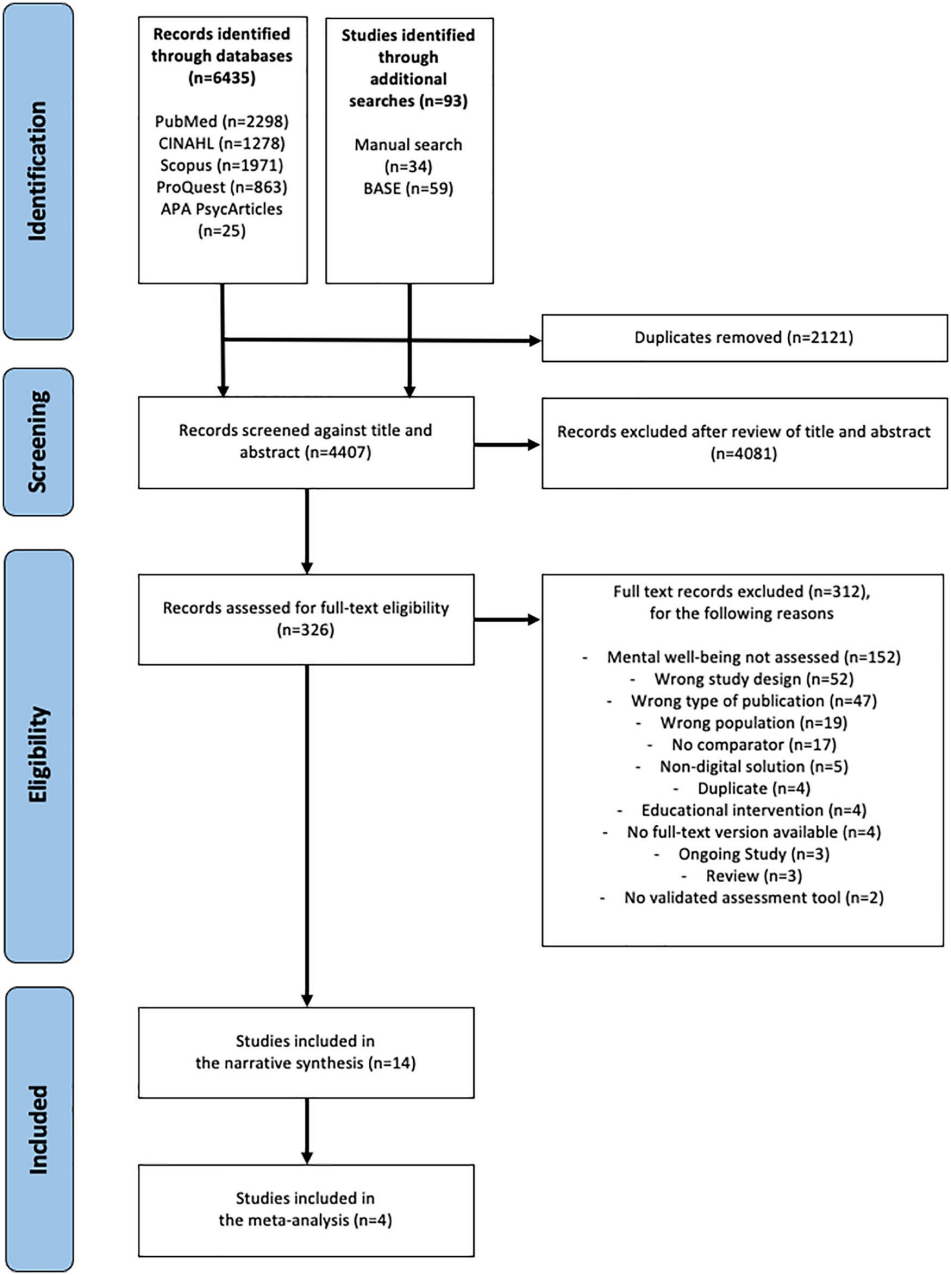
The search process of this systematic review of effectiveness of digital solutions improving healthcare professionals’ mental well-being, including inclusion criteria and study selection, is illustrated in accordance with the PRISMA 2020 statement. (Page et al., 2021). The PubMed, CINAHL, Scopus, Pro-Quest and APA PsycArticles databases were reviewed for randomised controlled trials and quasi-experimental studies published at any point prior to the 26th of October 2021.

The PRISMA statement (Page et al., 2021) was applied for reporting the selection process of the studies (Figure 1). The Covidence software (v2422) (https://www.covidence.org/terms/) was utilised to manage the identified references. The identified titles and abstracts were screened independently by two reviewers (TI, MM), who then assessed relevant full-text articles for eligibility. The two reviewers resolved any disagreements by reaching a consensus.

### Quality appraisal

The methodological quality of the identified studies was critically appraised by two independent reviewers (TI, MM) using JBI critical appraisal checklists (Tufanaru et al., 2020). The reviewers responded to each question by choosing either ‘Yes’ (Y), ‘Unclear’ (U), ‘No’ (N) or ‘Not Applicable’ (NA). Every ‘Yes’ answer was worth 1 point, whereas the other answers were worth 0 points. The quality of a study was determined as fair (F) if less than 50% of the items received a response of ‘Yes’; moderate (M) if between 51% and 80% of the items received a response of ‘Yes’ and good (G) if over 80% of the items received a response of ‘Yes’.

### Data extraction

Two independent reviewers (TI, MM) extracted data from the included studies (Munn et al., 2014). The data extraction form (Supplemental Table S2) was pretested and used to chart information that was significant to the review questions (Tufanaru et al., 2020).

### Methodological quality

None of the identified studies was excluded due to methodological quality. All the randomised controlled trials (RCTs; Supplemental Table S3) received moderate scores. The studies lost points because the randomisation process remained unclear in one study (Vallières et al., 2016), blinding was NA in this kind of study design, the treatment groups differed in two studies (Kloos et al., 2019; Vallières et al., 2016), and the demographics of group participants were not specified in one study (Vallières et al., 2016).

Of the quasi-experimental studies, one (Motamed-Jahromi et al., 2017) was classified as demonstrating good, whereas the others demonstrated moderate methodological quality (Supplemental Table S4). In one study, the pre-test group contained non-participants that could not be distinguished from the intervention participants (Gracia Gozalo et al., 2019). As such, it was not possible to distinguish participants based on the other interventions they had received.

High dropout rates were noticed in two studies (Bolier et al., 2014; Doran et al., 2010). The main reasons why participants left the study were that they no longer worked in the organisation, were on maternity leave, as well as unknown (Doran et al., 2010). Younger age and lower work engagement were found to predict dropout by Bolier et al. (2014).

### Data synthesis and analysis

Narrative synthesis (Popay et al., 2006) was performed for each of the 14 included studies. In addition, four RCTs were included in the meta-analysis of work engagement (Bolier et al., 2014; Kloos et al., 2019; Sasaki et al., 2021; Vallières et al., 2016). Each of these studies used the same scale, that is, Utrecht Work Engagement Scale (UWES; Schaufeli et al., 2002). There was no similar consistency in other outcomes and scales. For the meta-analysis, the separately reported subgroups were combined in two of the four studies: in one study (Sasaki et al., 2021) two intervention groups were combined; and in the second study (Vallières et al., 2016) two control groups were combined. This combination was performed using formulae in the Cochrane Handbook (Higgins and Green, 2011) to combine the sample sizes, means and standard deviations of the subgroups.

The meta-analysis was performed using JBI SUMARI (Munn et al., 2019). As work engagement was measured using two different Likert-scales (0–5 or 1–6), we used standardised mean difference (SMD) to compare the intervention and control groups. Cohen’s *d* values were used as a measure of SMD. Cohen’s *d* value is calculated by dividing the difference between mean scores of the intervention and control groups by the pooled standard deviation for the two groups. Cohen’s *d* value of 0.2 = small; 0.5 = medium and 0.8 = large effect (Cohen, 1992). In this study, negative Cohen’s *d* values meant that the control group showed higher work engagement scores than the intervention group, whereas a positive Cohen’s *d* value meant that the intervention group had higher work engagement scores than the control group. Cohen’s *d* values with 95% confidence intervals (CIs) were presented using a forest plot.

The meta-analysis utilised a random-effects model to pool the overall effect estimate, which assumed that the effect of the intervention was not identical in different study populations. In a random-effects model, each study is weighted by the inverse of its variance plus the between-study variance. We assessed the heterogeneity of the studies using the *I*^2^ statistic, and the statistical significance of heterogeneity was tested using the chi-square test (Cochran Q test); *I*^2^ values range from 0% to 100% to reflect the proportion of total variation across studies. *I*^2^ values of 25%, 50% and 75% describe low, moderate and high heterogeneity, respectively, across studies (Higgins et al., 2003).

## Results

A total of 4407 records were screened, whereas 326 studies were assessed for full-text eligibility; 312 studies were excluded because the inclusion criteria were not met (Figure 1). A total of 14 studies (Bolier et al., 2014; Doran et al., 2010; Dutton and Kozachik, 2020; Engström et al., 2009; Gracia Gozalo et al., 2019; Guo et al., 2020; Hersch et al., 2016; Hwang and Jo, 2019; Jakel et al., 2016; Kloos et al., 2019; Motamed-Jahromi et al., 2017; Sasaki et al., 2021; Vallières et al., 2016; van der Meer et al., 2020) were included in this systematic review (Supplemental Table S2), with four of these studies pooled in a meta-analysis (Bolier et al., 2014; Kloos et al., 2019; Sasaki et al., 2021; Vallières et al., 2016).

### Characteristics of the studies and participants

Of the 14 included studies, eight were RCTs and six were quasi-experimental studies. The studies were published between 2009 and 2021. They were conducted in various healthcare settings in Europe, North America, Asia, the Middle East and Africa.

The studies included 3000 participants at the baseline measurement point. Sample sizes ranged from 22 to 949 participants per study. Most of the participants were nurses, nursing assistants and nursing staff. In addition, the samples included community health workers, allied health professionals, physicians, paramedics and ambulance drivers. The total loss of participants between the baseline and follow-up phase was 591 (20%).

### Characteristics of digital interventions

The included research applied either personal mental well-being (*n* = 11) or work-related (*n* = 3) digital interventions. These interventions lasted between 1 and 18 months.

The personal mental well-being interventions included 2177 participants at the baseline measurement point. The 11 studies describing the effect of personal mental well-being interventions were published during the past 8 years. The interventions lasted between 1 and 3 months. Of these 11 studies, 5 evaluated mobile applications, such as positive psychology application Three Good Things or ABC Stress Management application (Guo et al., 2020; Hwang and Jo, 2019; Jakel et al., 2016; Sasaki et al., 2021; van der Meer et al., 2020). The studies included 1395 participants (nurses, physicians, paramedics and ambulance drivers). An additional four studies evaluated online programmes like web-based stress management programme BREATHE or online positive psychology intervention This Is Your Life (Bolier et al., 2014; Dutton and Kozachik, 2020; Hersch et al., 2016; Kloos et al., 2019). The 629 participants of these studies were all nursing staff. Moreover, a further two studies evaluated virtual message interventions, such as motivational content messages via WhatsApp or Telegram (Gracia Gozalo et al., 2019; Motamed-Jahromi et al., 2017). The 153 participants of these two studies were nurses, nursing assistants and physicians.

The work-related interventions included 823 participants at the baseline measurement point. These three studies (Doran et al., 2010; Engström et al., 2009; Vallières et al., 2016) were published between 2009 and 2016. The length of work-related digital interventions lasted between 5 and 18 months. The interventions involved evidence-based information resources (Doran et al., 2010), virtual healthcare software (Engström et al., 2009), and mobile human resource applications (Vallières et al., 2016).

### Mental well-being outcomes

The included studies evaluated 29 mental well-being outcomes using 31 scales. The most commonly evaluated positive mental well-being outcomes were work engagement (*n* = 4) (Bolier et al., 2014; Kloos et al., 2019; Sasaki et al., 2021; Vallières et al., 2016), job satisfaction (*n* = 4) (Doran et al., 2010; Engström et al., 2009; Kloos et al., 2019; Vallières et al., 2016) and well-being (*n* = 3) (Bolier et al., 2014; Hwang and Jo, 2019; Kloos et al., 2019). The most commonly evaluated negative outcomes were burnout (*n* = 2) (Gracia Gozalo et al., 2019; Jakel et al., 2016), depression (*n* = 2) (Bolier et al., 2014; Hwang and Jo, 2019), anxiety (*n* = 2) (Bolier et al., 2014; Hwang and Jo, 2019) and different kinds of stress (*n* = 5) (Dutton and Kozachik, 2020; Hersch et al., 2016; Hwang and Jo, 2019; Jakel et al., 2016). The most applied measure UWES was used in four studies.

### Effectiveness of digital solutions

The positive psychology application significantly improved the job performance subscales of job contribution (*F* = 6.425, *p* = 0.013), task performance (*F* = 29.252, *p* < 0.001) and facilitation of interpersonal relations (*F* = 17,682, *p* < 0.001) when the intervention group was compared to the control group (Guo et al., 2020). The stress management application significantly improved well-being (*F* = 4.07, *p* = 0.048), as well as decreased perceived (*F* = 3.33, *p* = 0.037) and occupational stress (*F* = 3.97, *p* = 0.050), when the intervention group was compared to the control group (Hwang and Jo, 2019). Moreover, two studies reported a significant self-efficacy improvement in the intervention group ((*F* = 5.058, *p* = 0.028; Guo et al., 2020); (*F* = 5.65, *p* = 0.021; Hwang and Jo, 2019)). The self-help application significantly improved resilience (*Z* = −4.191, *p* < 0.001) and decreased both post-traumatic negative cognitions (*Z* = −5.226, *p* < 0.001) and perceived lack of social support (*Z* = −2.74, *p* = 0.006; van der Meer et al., 2020) in the intervention group relative to the control group. The use of personal well-being mobile applications did not result in significant improvements in anxiety, depression, emotional labour (Hwang and Jo, 2019), burnout, compassion satisfaction, secondary traumatic stress (Jakel et al., 2016), or post-traumatic demoralisation syndrome (van der Meer et al., 2020).

The use of personal online stress management programmes significantly decreased nursing stress according to Dutton and Kozachik (2020) (*t* = 2.3, *p* < 0.026) and Hersch et al. (2016) (*t* = −2.95, *p* < 0.001). A workers’ health surveillance module significantly improved positive mental health (*F* = 3.46, *p* = 0.03) in the intervention group relative to the control group (Bolier et al., 2014). In the study by Kloos et al. (2019), an online positive psychology intervention significantly decreased job satisfaction in the control group (*F* = 4.54, *p* = 0.04). The tested personal well-being online interventions did not achieve significant impacts on general well-being (Bolier et al., 2014; Kloos et al., 2019), anxiety, depression (Bolier et al., 2014), coping with stress, nurses’ job satisfaction or work limitations (Hersch et al., 2016).

Personal virtual group messages significantly improved self-compassion (95% CI: 3–72, *p* = 0.001) and decreased the burnout subscale of emotional exhaustion (95% CI: −3 to 78, *p* = 0.012) among intervention group participants. This approach also significantly improved the mindfulness subscales of observation (95% CI: 3–13, *p* = 0.004), non-reactivity to inner experience (95% CI: 2–59, *p* = 0.035), decreased non-judging of inner experience (95% CI: −1 to 44, *p* = 0.002), and acting with awareness (95% CI: −2 to 31, *p* = 0.035) of the intervention group relative to the control group (Gracia Gozalo et al., 2019). Motamed-Jahromi et al. (2017) found that social networking messages significantly improved the quality of work-life (*p* < 0.001). Personal well-being virtual message solutions did not significantly influence the burnout subscales of depersonalisation, personal achievement or empathy or global mindfulness or its element of description (Gracia Gozalo et al., 2019).

Work-related internet access to information resources via a personal digital assistant (PDA) significantly improved job satisfaction (*F* = 15.4, *p* < 0.001) in the intervention group, but primarily in a long-term care setting (Doran et al., 2010). Work-related digital interventions did not significantly impact job satisfaction (Engström et al., 2009; Vallières et al., 2016), psychosomatic health (Engström et al., 2009), motivation or perceived supervision (Vallières et al., 2016).

**Figure 2.**
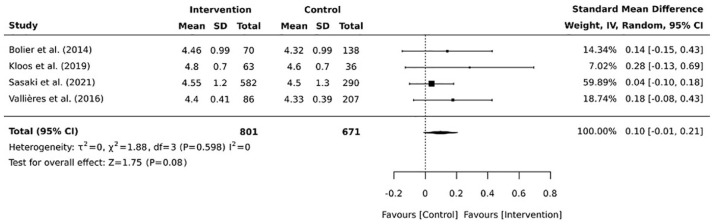
Four RCTs (Bolier et al., 2014; Kloos et al., 2019; Sasaki et al., 2021; Vallières et al., 2016) focusing on digital solutions improving healthcare professionals’ mental well-being were included in the meta-analysis of work engagement. The meta-analysis was performed using JBI SUMARI (Munn et al., 2019) in April 2022. Cohen’s *d* values with 95% confidence intervals (CIs) were presented using a forest plot to compare the effects of digital and non-digital solutions for supporting the work engagement of healthcare professionals. As shown in the figure, the digital interventions had little to no impact on work engagement.

The four RCTs (Bolier et al., 2014; Kloos et al., 2019; Sasaki et al., 2021; Vallières et al., 2016) that evaluated work engagement were pooled in a meta-analysis. Of these RCTs, three (Bolier et al., 2014; Kloos et al., 2019; Sasaki et al., 2021) evaluated personal digital mental well-being solutions, whereas one study (Vallières et al., 2016) evaluated work-related digital solutions. As shown in Figure 2, the overall effect estimate was small (*d* = 0.10; 95% CI: −0.01 to 0.21), which indicates that the digital interventions had little to no impact on work engagement. The *I*^2^ value was 0% (*p* = 0.598), which indicates that the RCTs showed low heterogeneity.

## Discussion

This systematic review has provided evidence that personal digital mental well-being solutions may have the potential to improve nurses’ and healthcare professionals’ mental well-being as well as quality of working life (Motamed-Jahromi et al., 2017) and job performance (Guo et al., 2020). Moreover, these solutions can decrease nursing stress (Dutton and Kozachik, 2020; Hersch et al., 2016). Notably, Ye (2021) stated that digital solutions are an innovative opportunity to promote well-being and work performance of nurses and healthcare professionals, as well as quality of care.

Similar to the research by Ackerman et al. (2018), the presented results reveal that well-being outcomes are related to meaning, purpose, strengths, values, relationships, social support, gratitude, spirituality, self-esteem and self-efficacy. The mental well-being was evaluated through numerous positive and negative outcomes and various scales. The studies included in this review could not, however, demonstrate the effectiveness of digital solutions on certain negative outcomes. This may be explained by the negative outcome variable already showing a low value at the baseline measurement point, which could make it almost impossible to demonstrate significant positive effects (Hwang and Jo, 2019). Any variation in our findings may be due to variability in the scales used for a single outcome variable; in other words, the findings obtained from different scales cannot be directly compared. Therefore, we recommend that the outcome variables of well-being should be carefully chosen in future research. More specifically, well-being research should focus on either positive or negative outcome variables, or one specific outcome, and employ specific validated scales.

Even though the results of our meta-analysis did not support the effectiveness of digital solutions, they nevertheless indicated that digital solutions might promote the work engagement of nurses and healthcare professionals. As Yildiz and Yildiz (2022) concluded, the work environment must enable work engagement and job satisfaction to maintain a sustainable healthcare system. In this way, nurse and healthcare managers should promote a work atmosphere, which provides high levels of organisational justice, perceived support, trust and empowerment.

The only significant result in the work-related studies was influenced by the fact that PDAs are considered the most user-friendly device and easiest to carry around (Doran et al., 2010). According to Nguyen et al. (2021), the healthcare sector has a critical need for digital solutions that enable professionals to better organise, manage and access the information they need to make effective clinical decisions. Hence, digital solutions should provide nurses with cost-effective and easy-to-use tools. Poor nurse well-being will negatively impact the healthcare system, that is, decreased quality of care, increased costs, and system inefficiency. Future research is needed to develop work-related digital solutions that can address these complexities.

### Limitations

We may have excluded some relevant studies by limiting the search to research published only in English. Ideally, evidence about the effectiveness of digital solutions should come from high-quality RCTs, but this review also included numerous quasi-experimental studies (Tufanaru et al., 2015). It is noteworthy that the quality of evidence concerning the effectiveness of digital solutions was reduced by variation in outcome variables and applied scales, small sample sizes, high dropout rates, short intervention durations and the lack of long-term monitoring. Moreover, a relatively small set of studies was included in the meta-analysis, and the small sample sizes may have reduced the statistical power of the calculations used to obtain intervention effects.

## Conclusions

Based on the results of this systematic review, personal mobile applications, online programmes and virtual message interventions, and work-related digital solutions have been used to improve nurses’ and healthcare professionals’ mental well-being. The results showed that personal mental well-being digital solutions significantly affected nurses’ and healthcare professionals’ mental health, self-efficacy, general well-being, elements of mindfulness and self-compassion, resilience, quality of working life, job performance and nursing stress. They may have potential to improve work engagement of healthcare professionals.

There is still limited evidence regarding the impact of work-related digital solutions on the well-being, work-engagement, and job satisfaction of nurses and healthcare professionals. This topic should be researched more thoroughly through high-quality RCTs with larger sample sizes and specifically selected outcome variables and scales. It is also highly recommended to update the systematic review, as the topic is actively researched. Nurse managers should facilitate nurses to actively use digital solutions to promote their personal well-being. They have a crucial role in calling for more high-level research on the effects of work-related digital solutions on the well-being of all healthcare professionals.

**Key points for policy, practice and/or research**

- As mental health disorders become more common, innovative methods are needed to influence the mental well-being of nurses and healthcare professionals.
- Digital personal mental well-being solutions have potential to improve nurses’ and healthcare professionals’ mental well-being.
- Nurse managers should facilitate the utilisation of digital personal mental well-being solutions by nurses to actively improve their mental well-being.
- Nurse managers have a crucial role in calling for more high-level research on the effects of work-related digital solutions on the well-being of nurses and healthcare professionals.

## Supplemental Material

sj-pdf-1-jrn-10.1177_17449871241226914 – Supplemental material for The effectiveness of digital solutions in improving nurses’ and healthcare professionals’ mental well-being: a systematic review and meta-analysis Supplemental material, sj-pdf-1-jrn-10.1177_17449871241226914 for The effectiveness of digital solutions in improving nurses’ and healthcare professionals’ mental well-being: a systematic review and meta-analysis by Tiina Ilola, Mikael Malmisalo, Elina Laukka, Heli Lehtiniemi, Tarja Pölkki, Maria Kääriäinen, Hong-Gu He and Outi Kanste in Journal of Research in Nursing
