# Supplementary material for: The effectiveness of digital solutions in improving nurses’ and healthcare professionals’ mental well-being: a systematic review and meta-analysis
Source: J Res Nurs. 2024 May 20;29(2):97–109. doi: 10.1177/17449871241226914 (PMC11271664; doi:10.1177/17449871241226914)
Supplement: sj-pdf-1-jrn-10.1177_17449871241226914 – Supplemental material for The effectiveness of digital solutions in improving nurses’ and healthcare professionals’ mental well-being: a systematic review and meta-analysis [file sj-pdf-1-jrn-10.1177_17449871241226914.pdf]

Supplementary Table S1. Database searches of effectiveness of digital solutions improving healthcare professionals´ mental well-being.

Database	Search Query		Results
PubMed	1.	digi*[tw] OR electronic*[tw] OR mobile*[tw] OR virtual*[tw] OR online*[tw] OR internet*[tw] OR "Internet"[Mesh] OR web-based[tw] OR smartphone*[tw] OR ”cell phone*”[tw] OR “Cell Phone”[Mesh] OR computer*[tw] OR computer-based[tw] OR computerized[tw] OR computerised[tw] OR “Computers, Handheld”[Mesh] OR "Computer Simulation"[Mesh] OR ehealth*[tw] OR e-health*[tw] OR mhealth*[tw] OR m-health*[tw] OR “mobile health”[tw] OR telehealth*[tw] OR “Telemedicine”[Mesh]	1,875,034
	2.	solution*[tw] OR application*[tw] OR app[tw] OR apps[tw] OR device*[tw] OR technolog*[tw] OR software*[tw] OR "Software"[Mesh] OR platform*[tw]	3,296,903
	3.	(#1 AND #2) OR “Mobile Applications”[Mesh] OR "Digital Technology"[Mesh] OR "Internet-Based Intervention"[Mesh]	559,506
	4.	well-being[tw] OR wellbeing[tw] OR wellness*[tw] OR satisfaction*[tw] OR ”Personal Satisfaction”[Mesh] OR ”Job Satisfaction”[Mesh] OR comfort*[tw] OR “positive experience*”[tw] OR "Health Promotion"[Mesh]	455,883
	5.	psych*[tw] OR mental*[tw] OR “Mental Health”[Mesh] OR "Psychological Phenomena"[Mesh]	3,474,961
	6.	#4 AND #5	221,439
	7.	”healthcare professional*”[tw] OR ”health care professional*”[tw] OR “health professional*”[tw] OR ”healthcare personnel*”[tw] OR ”health care personnel*”[tw] OR “health personnel*”[tw] OR “Health Personnel”[Mesh] OR ”healthcare worker*”[tw] OR ”health care worker*”[tw] OR “health worker*”[tw] OR “healthcare provider*”[tw] OR “health care provider*”[tw] OR “health provider*”[tw] OR nurse*[tw] OR ”nursing staff*”[tw] OR physician*[tw] OR doctor*[tw] OR clinician*[tw] OR ”medical staff*”[tw]	1,671,669
	8.	intervention*[tw] OR program*[tw] OR project*[tw] OR tool*[tw] OR questionnaire*[tw] OR measur*[tw] OR scale*[tw] OR psychometric*[tw] OR efficien*[tw] OR effect*[tw] OR impact*[tw] OR assess*[tw] OR evaluat*[tw] OR outcome*[tw] OR "Psychometrics"[Mesh] OR "Surveys and Questionnaires"[Mesh] OR "Efficiency"[Mesh] OR "Evaluation Studies as Topic"[Mesh]	18,173,308
	9.	#3 AND #6 AND #7 AND #8	2,355
	10.	#9 AND english[Filter]	2,298
CINAHL	1.	digi* OR electronic* OR mobile* OR virtual* OR online* OR internet* OR (MH “Internet+”) OR web-based OR smartphone* OR ”cell phone*” OR (MH ”Cellular phone+”) OR computer* OR computer-based OR computerized OR computerised OR telehealth* OR (MH “Telehealth+”) OR ehealth OR mhealth OR e-health OR m-health OR ”mobile health”	602,538
	2.	solution* OR application* OR app OR apps OR device* OR technolog* OR software* OR (MH “Software+”) OR platform*	950,417
	3.	#1 AND #2	177,764
	4.	(MH “Mobile Applications+”) OR (MH “Digital Technology+”)	13,899
	5.	#3 OR #4	179,798
	6.	wellbeing OR well-being OR wellness OR (MH “Wellness”) OR satisfaction* OR ”Personal Satisfaction” OR (MH “Personal Satisfaction+) OR ”Job Satisfaction” OR comfort* OR “positive experience*” OR (MH “Quality of Life+”)	246,556

	7.	psych* OR mental* OR (MH ”Mental Health+”) OR (MH "Psychological Well-Being")	1,054,412
	8.	#6 AND #7	106,506
	9.	”healthcare professional*” OR ”health care professional*” OR “health professional*” OR ”healthcare personnel*” OR ”health care personnel*” OR “health personnel*”OR (MH ”Health Personnel+”) OR ”healthcare worker*” OR ”health care worker*” OR “health worker*” OR nurse* OR ”nursing staff*” OR physician* OR doctor* OR clinician* OR ”medical staff”	1,247,918
	10.	intervention* OR program* OR project* OR tool* OR questionnaire* OR measur* OR scale* OR psychometric* OR efficien* OR effect* OR impact* OR assess* OR evaluat* OR outcome* OR (MH ”Intervention Trials”) OR (MH ”Program Evaluation+”) OR (MH ”Outcome Assessment+”) OR (MH ”Outcomes (Healthcare)+”) OR (MH ”Research Instruments+”)	4,202,023
	11.	#5 AND #8 AND #9 AND #10	1,299
	12.	# 11 AND (Limiters - English Language)	1,278
Scopus	1.	digi* OR electronic* OR mobile* OR virtual* OR online* OR internet* OR web-based OR ”mobile phone*” OR smartphone* OR ”cell phone*” OR ”mobile tablet*” OR iPad* OR computer* OR computer-based OR computerized OR computerised OR ehealth* OR e-health* OR mhealth* OR m-health* OR telehealth* OR tele-health* OR telemedicine* OR “mobile health”	30,965,475
	2.	solution* OR application* OR app OR apps OR device* OR technolog* OR software* OR platform*	38,692,526
	3.	#1 AND #2	22,063,371
	4.	wellbeing OR well-being OR wellness OR satisfaction* OR ”Personal Satisfaction” OR ”Job Satisfaction” OR comfort* OR “positive experience*”	2,203,437
	5.	psych* OR mental*	8,524,050
	6.	#4 AND #5	1,258,071
	7.	“healthcare professional*” OR ”health care professional*” OR “health professional*” OR ”healthcare personnel*” OR ”health care personnel*” OR “health personnel*” OR ”healthcare worker*” OR ”health care worker*” OR “health worker*” OR “healthcare provider*” OR “health care provider*” OR “health provider*” OR nurse* OR ”nursing staff*” OR physician* OR doctor* OR clinician* OR ”medical staff*”	4,893,509
	8.	intervention* OR program* OR project* OR tool* OR questionnaire* OR measur* OR scale* OR psychometric* OR efficien* OR effect* OR impact* OR assess* OR evaluat* OR outcome*	57,411,009
	9.	#3 AND #6 AND #7 AND #8	163,817
	10.	TITLE-ABS-KEY (#9) AND (LIMIT-TO (LANGUAGE , "English"))	1,971
Proquest	1.	digi* OR electronic* OR mobile* OR virtual* OR online* OR internet* OR web-based OR ”mobile phone*” OR smartphone* OR ”cell phone*” OR ”mobile tablet*” OR iPad* OR computer* OR computer-based OR computerized OR computerised OR ehealth* OR e-health* OR mhealth* OR m-health* OR telehealth* OR tele-health* OR telemedicine* OR “mobile health”	19,325,553
	2.	solution* OR application* OR app OR apps OR device* OR technolog* OR software* OR platform*	20,619,604
	3.	1 AND 2	6,648,543
	4.	wellbeing OR well-being OR wellness OR satisfaction* OR ”Personal Satisfaction” OR ”Job Satisfaction” OR comfort* OR “positive experience*”	1,318,146

	5.	psych* OR mental*	2,109,375
	6.	4 AND 5	135,494
	7.	”healthcare professional*” OR ”health care professional*” OR “health professional*” OR ”healthcare personnel*” OR ”health care personnel*” OR “health personnel*” OR ”healthcare worker*” OR ”health care worker*” OR “health worker*” OR “healthcare provider*” OR “health care provider*” OR “health provider*” OR nurse* OR ”nursing staff*” OR physician* OR doctor* OR clinician* OR ”medical staff*”	1,986,392
	8.	intervention* OR program* OR project* OR tool* OR questionnaire* OR measur* OR scale* OR psychometric* OR efficien* OR effect* OR impact* OR assess* OR evaluat* OR outcome*	28,372,565
	9.	3 AND 6 AND 7 AND 8	864
	10.	9 AND Applied filters: English	863
APA Psyc-Articles	1.	digi* OR electronic* OR mobile* OR virtual* OR online* OR internet* OR web-based OR smartphone* OR ”cell phone*” OR computer* OR computer-based OR computerized OR computerised OR telehealth* OR ehealth OR mhealth OR e-health OR m-health OR ”mobile health”	18,046
	2.	solution* OR application* OR app OR apps OR device* OR technolog* OR software* OR platform*	20,353
	3.	#1 AND #2	2,913
	4.	wellbeing OR well-being OR wellness OR satisfaction* OR ”Personal Satisfaction” OR ”Job Satisfaction” OR comfort* OR “positive experience*”	14,827
	5.	psych* OR mental*	370,479
	6.	#4 AND #5	14,477
	7.	”healthcare professional*” OR ”health care professional*” OR “health professional*” OR ”healthcare personnel*” OR ”health care personnel*” OR “health personnel*” OR ”healthcare worker*” OR ”health care worker*” OR “health worker*” OR nurse* OR ”nursing staff*” OR physician* OR doctor* OR clinician* OR ”medical staff”	20,838
	8.	intervention* OR program* OR project* OR tool* OR questionnaire* OR measur* OR scale* OR psychometric* OR efficien* OR effect* OR impact* OR assess* OR evaluat* OR outcome*	162,153
	9.	#3 AND #6 AND #7 AND #8	25
	10.	# 9AND (Narrow by Language: - English)	25

Supplementary Table S2. Summary of the reviewed studies (n=14) of digital solutions improving healthcare professionals´ mental well-being. The PubMed, CINAHL, Scopus, Pro-Quest, and APA PsycArticles databases were reviewed for randomised controlled trials and quasi-experimental studies published at any point prior to the 26th of October 2021.

Study, year, country	Study design	Setting	Participants	Baseline (n)	Completors (n)	Duration (months)	Digital intervention	Control group and intervention	Scale	Outcome results
Bolier et al., 2014, the Netherlands	Cluster-randomized controlled trial with two groups	Large academic medical centre	Nurses and allied health professionals	366	208	1,5	Personal workers' health surveillance (WHS) module including personal online well-being interventions (Psyfit, Colour Your Life, Strong at Work, Don't Panic Online, Drinking Less) and personalised advice.	Waitlisted control group (WL), no intervention	Brief Symptom Inventory (BSI)	Anxiety: No difference
									Brief Symptom Inventory (BSI)	Depression: No difference
									The Mental Health Continuum - Short Form (MHC-SF)	Positive mental health: Improved (s)
									The WHO Well-being Scale (WHO-5)	Well-being: No difference
									The Utrecht Work Engagement Scale (UWES-9)	Work engagement*: Improved (s)
Doran et al., 2010, Canada	Longitudinal, pre- and post-test design	Acute care, long-term-care, home care, and	Registered nurses and registered practical nurses	488	223	12	Work-related access to high-quality evidence-based information	No control group	Global measure of work satisfaction	Job satisfaction: Improved (s) for PDA users in long-term care.

		correlational organisations					resources via mobile technology (personal digital assistants (PDAs) and tablet PCs).			
Dutton & Kozachik, 2020, United States	Quality improvement project including a single group, pre- and post-test design	Subacute rehabilitation unit at a community hospital	Nurses and nursing assistants	31	31	2	Personal web-based stress management program (BREATHE).	No control group	Nurse Stress Scale (NSS)	Nursing stress: Improved (s)
Engström et al., 2009, Sweden	Quasi-experimental design	Home care	Nursing assistants	22	18	5	Work-related OLD@HOME, the Virtual Health software and mobile technology (tablet PC, laptop or personal digital assistant (PDA).	Comparison group, traditional documentation	The Satisfaction with Work Questionnaires (SWQ)	Job satisfaction: No difference<br>Psychosomatic health: No difference
Gracia Gozalo et al., 2019, Spain	Longitudinal study with a pre- and post-test intervention design	Intensive Care Unit of a tertiary hospital	Physicians, nurses, and nursing assistants	53	32	2	Personal weekly virtual group (WhatsApp) messages including guided meditation and motivational content.	No control group	The Maslach Burnout Inventory (MBI)<br>Jefferson Scale of Emphaty (JSE)	Burnout/Emotional exhaustion: Decreased (s)<br>Burnout/Depersonalisation: No difference<br>Burnout/Personal achievement: No difference<br>Emphaty: No difference

									Five Facets of Mindfulness Questionnaire (FFMQ)	Midfulness/Global: No difference<br>Midfulness/Observation: Improved (s)<br>Midfulness/Description: No difference<br>Midfulness/Acting with awareness: Decreased (s)<br>Midfulness/Non-judging of inner experience: Decreased (s)<br>Midfulness/Non-reactivity to inner experience: Improved (s)
									Self-Compassion Scale (SCS)	Self-compassion/Global: Improved (s)<br>Self-compassion/Self-kindness: Improved (s)<br>Self-compassion/Common or shared humanity: Improved (s)<br>Self-compassion/Mindfulness: Improved (s)
Guo et al., 2020, China	A randomised controlled trial	Tertiary general hospital	Registered nurses with a MBI-GS score higher than 1,5	102	73	1,5	Personal positive psychology mobile application (Three Good Things).	Control group, no intervention	Job Performance Scale (JPS)	Job performance: Improved (s)
									General Self-efficacy Scale (GSS)	Self-efficacy: Improved (s)
Hersch et al., 2016, United States	Randomised controlled trial	Suburban and metropolitan hospitals	Nurses	104	104	3	Personal web-based stress management	Control group, no intervention	Nurse Stress Scale (NSS)	Nursing stress: Improved (s)
									Coping with stress	Coping with stress: No difference

							program (BREATHE).		Work Limitations Questionnaire (WLQ)	Work limitations: No difference
									Nurses' Job Satisfaction Scale	Nurses' job satisfaction: No difference
Hwang & Jo, 2019, Korea	Randomised controlled trial	College hospitals and the surrounding metropolitan area.	Nurses	60	56	1	Personal mental health mobile application.	Control group, no intervention	General Anxiety Disorder Scale (GAD-7)	Anxiety: No difference
									Patient Health Questionnaire: Depression (PHQ-9)	Depression: No difference
									The Korean-Emotional Labor scale	Emotional labor: No difference
									Korean Occupational Stress Scale (KOSS)	Occupational stress: Decreased (s)
									Perveived Stress Scale (PSS)	Perceived stress: Decreased (s)
									General Self-efficacy Scale (GSS)	Self-efficacy: Improved (s)
									The WHO Well-being Scale (WHO-5)	Well-being: Improved (s)
Jakel et al., 2016, United States	Prospective, quasi-experimental	Inpatient oncology unit	Nurses	25	25	1,5	Personal provider resilience	Control group, no intervention	Professional Quality of Life (ProQOL 5)	Burnout: No difference<br>Compassion satisfaction: No difference

	design with a longitudinal approach						mobile application (PRMA).			Secondary traumatic stress: No difference
Kloos et al., 2019, the Netherlands	Cluster-randomised controlled design	The units for physically frail older adults of four nursing homes	Members of nursing staff	128	107	2	Personal online positive psychology intervention (This Is Your Life).	Control group, no intervention	The Mental Health Continuum - Short Form (MHC-SF)	General well-being: No difference
									Maastricht Job Satisfaction Scale for Healthcare (MAZ-GZ)	Job satisfaction: Stable in the intervention group, but decreased in the control group (s)
									The Utrecht Work Engagement Scale (UWES-9)	Work engagement*: No difference
Motamed-Jahromi et al., 2017, Iran	Quasi-experimental study design with a pre- and post-test design	Two hospitals	Nurses	100	100	3	Personal positive content and messages via networking application (Telegram).	Control group, paper-based information (positive thinking books)	Quality of Work Life questionnaire (QWL)	Quality of working life: Improved (s)
Sasaki et al., 2021, Vietnam	Three-arm randomised controlled trial	A large public tertiary hospital	Registered nurses	949	949	1,5	Personal stress management mobile application (ABC Stress Management); two groups.	Control group, no intervention	The Utrecht Work Engagement Scale (UWES-9)	Work engagement*: No difference
Vallières et al., 2016, Sierra Leone	Three-arm randomised longitudinal cohort design	Community healthcare	Health workers	313	293	18	Work-related mHealth mobile application (MOTECH Suite) and mobile technology.	Two control groups who did not use mobile technology and/or the app.	Minnesota Satisfaction Questionnaire (MSQ)	Job satisfaction: No difference
									Motivation: Volunteer Functions	Motivation: No difference

									Inventory (VFI)	
									Perceived Supportive Supervision Scale (PSSS)	Perceived supervision: No difference
									The Utrecht Work Engagement Scale (UWES)	Work engagement*: No difference
van der Meer et al., 2020, the Netherlands	Randomized controlled trial	Multiple hospitals and ambulance regions	Healthcare professionals (nurses, physicians, paramedics and ambulance drivers) who indicated at least one post-traumatic stress symptom	259	183	1	Personal self-help mobile application to reduce trauma-related symptoms.	Control group, no intervention	PTSD checklist for DSM-5 (PCL-5)	Post-traumatic demoralisation syndrome (PTDS) symptoms: No difference
									Peritraumatic Cognitions Inventory (PTCI)	Post-traumatic negative cognitions: Decreased (s)
									Resilience Evaluation Scale (RES)	Resilience: Improved (s)
									Social Support List (SSL-6)	Perceived lack of social support: Decreased (s)

Abbreviations: s = significant; * = included in the meta-analysis

Supplementary Table S3. Critical appraisal of the eligible randomised controlled trials of digital solutions improving healthcare professionals´ mental well-being using the JBI Critical Appraisal Checklist for Randomized Controlled Trials (Tufanaru et al., 2020).

Question		Bolier et al., 2014	Guo et al., 2020	Hersch et al., 2016	Hwang & Jo, 2019	Kloos et al., 2019	Sasaki et al., 2021	Vallières et al., 2016	van der Meer et al., 2020	Total (n)	Total (%)
Q1	Was true randomisation used for assignment of participants to groups?	Y	Y	Y	Y	Y	Y	U	Y	7	88%
Q2	Was allocation to treatment groups concealed?	NA	NA	NA	NA	NA	NA	NA	NA	0	0%
Q3	Were treatment groups similar at the baseline?	Y	Y	Y	Y	N	Y	N	Y	6	75%
Q4	Were participants blind to treatment assignment?	NA	NA	NA	NA	NA	NA	NA	NA	0	0%
Q5	Were those delivering treatment blind to treatment assignment?	NA	NA	NA	NA	NA	NA	NA	NA	0	0%
Q6	Were outcomes assessors blind to treatment assignment?	NA	NA	NA	NA	NA	NA	NA	NA	0	0%
Q7	Were treatment groups treated identically other than the intervention of interest?	Y	Y	Y	Y	Y	Y	Y	Y	8	100%
Q8	Was follow up complete and if not, were differences between groups in terms of their follow up adequately described and analysed?	Y	Y	Y	Y	Y	Y	Y	Y	8	100%
Q9	Were participants analysed in the groups to which they were randomised?	Y	Y	Y	Y	Y	Y	Y	Y	8	100%
Q10	Were outcomes measured in the same way for treatment groups?	Y	Y	Y	Y	Y	Y	Y	Y	8	100%
Q11	Were outcomes measured in a reliable way?	Y	Y	Y	Y	Y	Y	Y	Y	8	100%
Q12	Was appropriate statistical analysis used?	Y	Y	Y	Y	Y	Y	Y	Y	8	100%
Q13	Was the trial design appropriate, and any deviations from the standard RCT design individual randomisation, parallel groups) accounted for in the conduct and analysis of the trial?	Y	Y	Y	Y	Y	Y	Y	Y	8	100%
	Total score (n)	9	9	9	9	8	9	7	9		
	Total score (%)	69%	69%	69%	69%	62%	69%	54%	69%		
	Quality	M	M	M	M	M	M	M	M		

Abbreviations: M=moderate; N=no; NA=not applicable; Y=yes.

Supplementary Table S4. Critical appraisal of the eligible quasi-experimental trials of digital solutions improving healthcare professionals´ mental well-being using the JBI Critical Appraisal Checklist for Quasi-Experimental Studies (non-randomised experimental studies) (Tufanaru et al., 2020).

Question		Doran et al., 2010	Dutton & Kozachik, 2020	Engström et al., 2009	Gracia Gozalo et al., 2019	Jakel et al., 2016	Motamed-Jahromi et al., 2017	Total (n)	Total (%)
Q1	Is it clear in the study what is the 'cause' and what is the 'effect' (i.e. there is no confusion about which variable comes first)?	Y	Y	Y	Y	Y	Y	6	100%
Q2	Were the participants included in any comparisons similar?	Y	Y	Y	N	Y	Y	5	83%
Q3	Were the participants included in any comparisons receiving similar treatment/care, other than the exposure or intervention of interest?	NA	NA	NA	NA	NA	NA	0	0%
Q4	Was there a control group?	N	N	Y	N	Y	Y	3	50%
Q5	Were there multiple measurements of the outcome both pre and post the intervention/exposure?	Y	Y	Y	Y	Y	Y	6	100%
Q6	Was follow up complete and if not, were differences between groups in terms of their follow up adequately described and analysed?	Y	Y	Y	Y	Y	Y	6	100%
Q7	Were the outcomes of participants included in any comparisons measured in the same way?	Y	Y	Y	Y	Y	Y	6	100%
Q8	Were outcomes measured in a reliable way?	Y	Y	Y	Y	Y	Y	6	100%
Q9	Was appropriate statistical analysis used?	Y	N	N	N	N	Y	2	33%
	Total score (n)	7	6	7	5	7	8		
	Total score (%)	78%	67%	78%	56%	78%	89%		
	Quality	M	M	M	M	M	G		

Abbreviations: G=good; M=moderate; N=no; NA=not applicable; Y=yes.
